# Application of Loop-Mediated Isothermal Amplification in an Early Warning System for Epidemics of an Externally Sourced Plant Virus

**DOI:** 10.3390/plants8050139

**Published:** 2019-05-27

**Authors:** Benjamin Congdon, Paul Matson, Farhana Begum, Monica Kehoe, Brenda Coutts

**Affiliations:** Sustainability and Biosecurity, Department of Primary Industries and Regional Development, 3 Baron-Hay Court, Kensington 6151, Australia; paul.matson@dpird.wa.gov.au (P.M.); farhana.begum@dpird.wa.gov.au (F.B.); monica.kehoe@dpird.wa.gov.au (M.K.); brenda.coutts@dpird.wa.gov.au (B.C.)

**Keywords:** viruliferous aphid, diagnostic, insect trapping, Myzus persicae, turnip yellows virus, disease management, insecticide, decision support

## Abstract

Restricting *Turnip yellows virus* (TuYV) spread in canola (*Brassica napus*) crops often relies upon the application of systemic insecticides to protect young vulnerable plants from wide-scale green-peach aphid (GPA; *Myzus persicae*) colonization and subsequent virus infection. For these to be applied at the optimal time to ensure they prevent epidemics, growers would need to be forewarned of incoming viruliferous aphid migration and colonization. This study was conducted to field validate a loop-mediated isothermal amplification (LAMP) protocol designed to detect TuYV in aphids caught on traps and develop an early warning system for TuYV epidemics. Double-sided yellow sticky traps were deployed at 30 sites sown with canola over a two-year period in the south-west Australian grainbelt. Using LAMP, the percentage (%) of trap sides with TuYV-carrying aphids was measured and related to TuYV infection incidence in the adjacent crop. When TuYV was detected in aphids on >30% trap sides in a six-week period from pre-emergence to GS15 (five-leaf stage), TuYV reached >60% crop incidence by GS30 (beginning of stem elongation). Whereas, TuYV detection in aphids on ≤15% trap sides during this period was associated with ≤6% TuYV incidence by GS30. Furthermore, when large numbers of aphids, including GPA, were caught during this period but no TuYV was detected in them, minimal TuYV spread (≤5%) occurred in the crop by GS30. Therefore, the LAMP TuYV protocol can be used in an early warning system for TuYV epidemics by providing detection of initial viruliferous aphid migration into a canola crop before they establish colonies throughout the crop and spread virus. This would enable proactive, non-prophylactic, and thereby more effective systemic insecticide applications to minimize seed yield and quality losses due to early season TuYV infection.

## 1. Introduction

The south-west Australian grain-growing region (grainbelt) experiences a Mediterranean-type climate consisting of a cool, wet growing season and hot, dry non-cropping period. Broadacre rainfed annual grain crops are grown throughout the early-autumn to late-spring growing season. Canola (*Brassica napus*, rapeseed cultivars with <30 µmol glucosinolate and <2% erucic acid seed contents), grown for its valuable oilseed, is the second most economically important crop behind wheat (*Triticum aestivum*) [1]. Canola also offers a range of weed and disease break opportunities for cereal production [2]. 

*Turnip yellows virus* (TuYV, Family *Luteoviridae*, Genus *Polerovirus*), is persistently transmitted by aphids (circulative, non-propagative), and the most widespread and economically damaging virus of grainbelt canola crops [3]. When reaching high incidences during the rosette growth phase (GS10 to 30 in the ‘BBCH’ decimal system) [4], TuYV can cause seed yield losses of >40%, decreases in oil content, and increases in erucic acid and glucosinolate contents [5,6,7,8]. The principal TuYV vector green-peach aphid (GPA; *Myzus persicae*, *Hemiptera: Aphididae*), is extremely effective at spreading the virus and difficult to control. GPA is a highly adaptable species facilitated by rapid transcriptional plasticity of genes contributing to its ability to colonize a wide host range thereby increasing its capacity to survive over the non-cropping period and arrive early in the growing season [9,10]. Once colonization is initiated, GPA spreads rapidly across large areas of canola transmitting TuYV at >90% efficiency [11]. Furthermore, GPA has developed an unrivalled insecticide resistance profile [12], including target site resistance to synthetic pyrethroids and carbamates, and metabolic resistance to organophosphates and neonicotinoids in Australia [13]. *Brevicoryne brassicae* (cabbage aphid, *Hemiptera: Aphididae*) also transmits TuYV but is considered to be of minor importance due to its inefficient transmission and dense vertical colonization, thereby limiting its virus spread to crop edges [11,14]. During the grainbelt non-cropping period, TuYV and GPA survive in reservoirs of volunteer or weed host plants, often in isolated damp locations such as roadside ditches and creeks [15]. Generally, the most important reservoir hosts are volunteer canola and wild radish (*Raphanus raphanistrum*), but many other broad-leafed weeds can provide reservoirs [3,16]. Following late-summer and early-autumn (February to April) rainfall events, further germination and growth of host plant species allows GPA colonies to increase and TuYV reservoirs to expand. Viruliferous GPA then migrate into canola crops, providing the initial infection foci for further spread [17].

Canola crops can be vulnerable to TuYV-induced losses throughout the rosette phase up until approximately stem elongation (~GS30). Significant losses from TuYV infection are unlikely after this point [7,8]. To protect seedlings from GPA colonization and the crop from wide-scale TuYV spread, neonicotinoids (Insecticide Resistance Action Committee [IRAC] group 4A) applied as a seed treatment, are widely adopted [18]. However, these do not protect plants throughout the entire vulnerable rosette phase [8]. Furthermore, efficacy of seed treatments can be reduced by environmental factors such as temperature and moisture stress [19,20] and substandard application, resulting in poor seed coverage [18]. Metabolic resistance to neonicotinoids *via* enhanced expression of the P450 CYP6CY3 gene has been identified in grainbelt GPA clones and could reduce the period in which plants are protected and magnify environmental factors and substandard seed coverage [18,21]. Therefore, application of a systemic foliar insecticide is often required to protect crops from TuYV during the rosette phase. Sulfoxaflor (IRAC group 4C - sulfoxamines) is currently the only registered insecticide that provides effective contact, translaminar and systemic GPA control [13,22,23], making it invaluable for TuYV control. However, target-site resistance to neonicotinoids (R81T mutation) that can confer cross-resistance to sulfoxaflor exists in holocyclic GPA populations in other world regions. The R81T mutation represents a significant biosecurity threat but could also evolve independently in Australian populations from misuse of sulfoxaflor [24,25,26]. To avoid or delay any potential future resistance issues, and effectively control TuYV, insecticides need to be utilized proactively and non-prophylactically. For this to occur, growers need early warnings of TuYV infection to make an informed insecticide application. 

TuYV detection in commercial canola crops has been limited to diagnosis (using molecular and serological techniques) in leaf samples from symptomatic plants. In these cases, the optimal time for a decisive insecticide application has passed, and the practical value of diagnosis is limited to retrospective advice. Recently, a reverse-transcription loop-mediated isothermal amplification (RT-LAMP) protocol was developed under laboratory conditions to detect TuYV-viruliferous aphids (taken directly from infected canola plants), amongst large numbers of non-viruliferous aphids and extracted from insect traps [27]. This protocol could enable TuYV detection in winged migratory aphids before they establish colonies throughout the crop and spread virus to high incidences, thus advising growers if and when to apply insecticide. However, it needs to be validated under field conditions where aphids commonly feed on intermediate plants before entering the crop and environmental conditions including moisture, temperature, humidity and light intensity may impact on sample quality and detection sensitivity. Furthermore, the presence of other aphid species carrying TuYV may produce an overestimation of risk, so the ability to identify the presence of GPA on traps may also be useful. A similar approach to obtain an assessment of infectivity of aphids migrating into cereal crops carrying *Barley yellow dwarf virus* (Family *Luteoviridae*, Genus *Luteovirus*) in aphids was developed using quantitative reverse-transcription polymerase chain reaction (qRT-PCR) [28]. Similarly, the numbers and relative percentages of aphids carrying *Citrus tristeza virus* (Family *Closteroviridae*; Genus, *Closterovirus*) assessed by nested RT-PCR, is used to explain high incidence and rapid spread during epidemic years [29]. These approaches rely on being able to catch a representative and sufficient number of migratory aphids from the field, and diagnostic protocols that are sensitive enough to detect virus in pooled aphid samples [28,30]. The advantages of LAMP in this context are its sensitivity, specificity and rapid result delivery. This paper describes a study conducted to: (i) develop and validate LAMP primers for GPA detection; (ii) field validate the use of LAMP of TuYV and GPA by testing aphids caught on yellow sticky traps; and (iii) develop an early warning system by examining the relationships between detection of virus-carrying aphids and crop TuYV incidence.

## 2. Results

### 2.1. GPA-Specific LAMP Protocol Development and Validation

Primer set GPA-FDS1 did not cross-react (no amplification) with DNA of any other of the seven aphid species tested. For all four repeat experiments, LAMP consistently detected a single GPA individually and at all dilutions with cowpea aphids in total DNA extractions (Table 1). Except for the 1/20 dilution which was significantly faster than the 1/100 dilution (*p* = 0.003), there was no significant difference in amplification time between any dilution. 

### 2.2. LAMP Protocol Field Validation

In 2017 and 2018, there was 92% and 93% congruence, respectively, between RT-LAMP and RT-PCR in TuYV detection in aphid trap samples. Of the aphid trap samples TuYV positive, 55% and 51% were also positive for GPA in 2017 and 2018, respectively. Although aphid numbers caught on the yellow sticky traps were generally higher on the trap side facing the prevailing wind, TuYV detections in aphids were equal on both sticky trap sides across all sites and both years and for any individual site.

### 2.3. Aphid Numbers, LAMP Detection and Virus Incidence

#### 2.3.1. 2017 Sites

**Figure 1:**
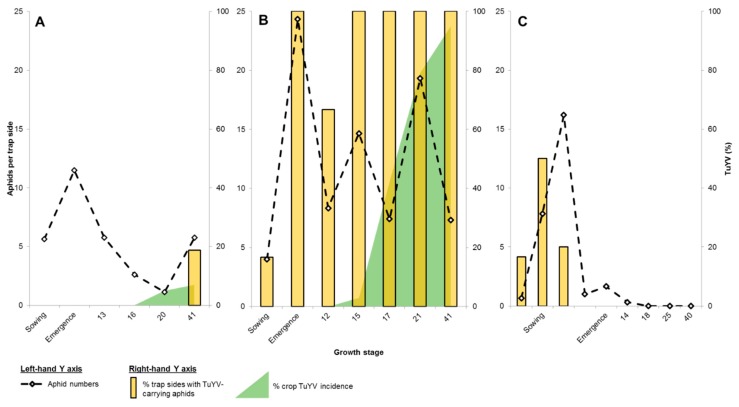
Aphid numbers, percentage (%) of trap sides with *Turnip yellows virus* (TuYV)-carrying aphids and canola (*Brassica napus*) crop virus incidence over the early period of the growing season at Coomalbidgup in 2017 (**A**) and 2018 (**B**), and at South Stirlings in 2018 (**C**). At each site, three double-sided yellow sticky traps were tied to the top of the fence and collected every two weeks. Aphids caught on each trap side were counted, pooled, homogenized, and the crude extract tested for TuYV by reverse transcription loop-mediated isothermal amplification. For canola crops, tip leaf samples of 100 to 200 plants tested for TuYV from ~GS12 (two-leaf stage; ‘BBCH’ decimal system, Lancashire et al 1991) by enzyme-linked immunosorbent assay.

**Figure 2:**
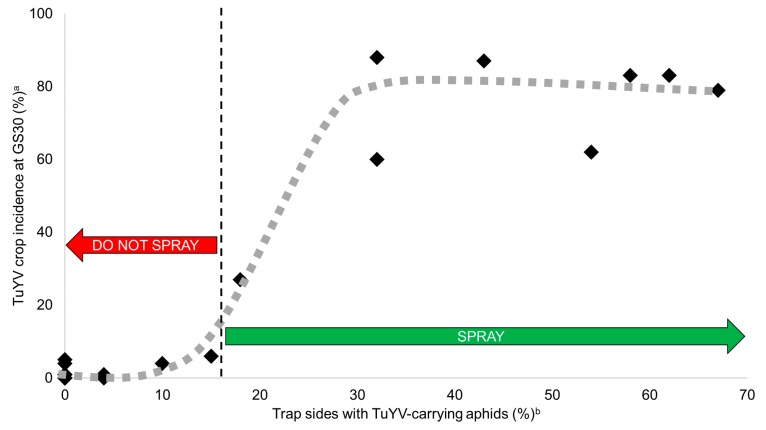
Relationship between canola (*Brassica napus*) crop *Turnip yellows virus* (TuYV) incidence at GS30 (beginning of stem elongation) and percentage (%) of trap sides with TuYV-carrying aphids deployed during a six-week period from pre-emergence until GS15 (five-leaf stage). Implication for insecticide application decision illustrated by red (do not spray) and green (spray) arrows. ^a^ Tip leaf samples of 100 to 200 canola plant tested for TuYV by enzyme-linked immunosorbent assay. Growth stages determined using the ‘BBCH’ decimal system [4]. ^b^ Yellow sticky traps deployed on top of fence adjacent to canola crop and collected every two weeks. Aphids caught on each trap side were pooled, homogenized, and the crude extract tested for TuYV by reverse transcription loop-mediated isothermal amplification.

TuYV was not detected in any broad-leaf weed samples (4120 plants tested across 14 sites) except for wild radish at Nunile and South Stirlings (both <1% of plants tested) and in 100% of the volunteer canola at Irish Town (Table 2). At all sites, aphids were regularly caught (2 to 9 per trap side) on sticky traps deployed around crop emergence to GS15 (e.g., Figure 1A). TuYV was detected in aphids on 32% of trap sides at Irish Town, 18% at Kojaneerup, 10% at Coomalbidgup, 4% at South Stirlings and Wellstead, but was not detected at the other nine sites (Figure 2). GPA were detected on 6 to 32% of trap sides at all sites except South Stirlings, Gairdner and Wellstead where no GPA were detected. Crop TuYV incidence at GS30 did not exceed 5% at any location except for Irish Town (60% crop infected) and Kojaneerup (27%). Crop TuYV incidences at GS75 were high (>50%) at Coomalbidgup (83%) and Gairdner (75%); moderate (20 to 50%) at Kojaneerup (47%), Wellstead (43%), Wongamine (41%), Jerramungup (38%), Kendenup (27%), Munglinup (27%), South Stirlings (25%), Mount Barker (23%) and Gibson (20%); and low (<20%) at Esperance Downs (12%) and Nunile (11%). 

#### 2.3.2. 2018 Sites

TuYV was not detected in any broad-leaf weed samples (979 plants tested across 11 sites) except for subterranean clover (<1%, *Trifolium subterraneum*) at Gibson. At Esperance Downs, Munglinup, Dalyup, Gibson, Coomalbidgup and Grass Patch, aphids were regularly caught on sticky traps (6 to 14 aphid per trap side) deployed around crop emergence to GS15 (e.g., Figure 1B). At these sites, TuYV was detected in aphids on 32 to 67% of trap sides. GPA were detected on 14 to 39% of trap sides. During the same period, few aphids (1 to 2 aphids per trap side) were caught at Bejoording, Nunile, Jerramungup, South Stirlings and Wongamine. Of these sites, TuYV was detected in aphids at Jerramungup only (15% of trap sides). GPA was detected on 15% trap sides at Jerramungup, 8% at Nunile and 4% at Bejoording. No aphids were caught at Coondle, Gairdner, Kendenup, Mount Barker or Tenterdon. There were high crop TuYV incidences at GS30 at Esperance Downs (88%), Munglinup (87%), Dalyup (83%), Gibson (83%), Coomalbidgup (79%) and Grass Patch (62%). Crop TuYV incidences reached 100% at each of these sites during flowering. There was minimal crop TuYV incidence at GS30 at Jerramungup (6%), and no TuYV detected at Bejoording, Coondle, Gairdner, Kendenup, Mount Barker, Nunile, South Stirlings, Tenterdon and Wongamine. Of these, there were high incidences at GS75 at Jerramungup (63%), and very low incidences (0 to 4%) at the rest. The crop at Gairdner was eliminated prior to flowering.

### 2.4. Predicting TuYV Epidemics

**Figure 3:**
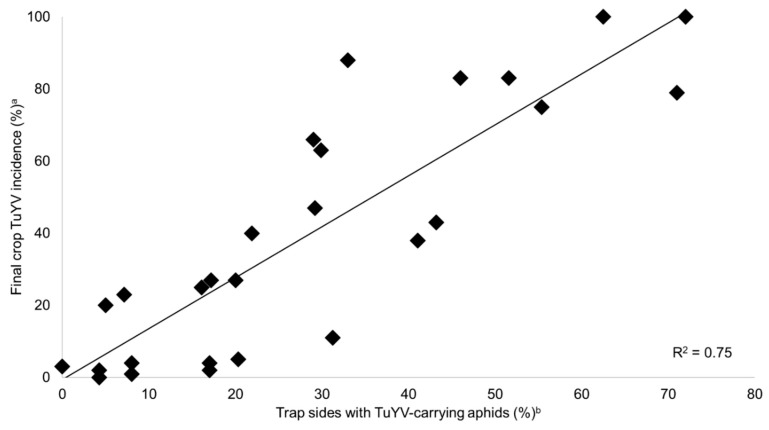
Relationship between final crop *Turnip yellows virus* (TuYV) incidence and percentage (%) of trap sides with TuYV-carrying aphids deployed in a four to twelve week period prior. ^a^ Final TuYV incidence data point used was at end of exponential spread or final sampling. Tip leaf samples from 100 to 200 individual canola plants tested for TuYV by enzyme-linked immunosorbent assay. ^b^ Three double-sided yellow sticky traps deployed and collected on edge of canola crop every two weeks during the four to twelve week period prior to final incidence. Aphids caught on each trap side were pooled, homogenized, and the crude extract tested for TuYV by reverse transcription loop-mediated isothermal amplification.

When incorporated individually into a linear regression with crop incidence at GS30 as the dependent variable, percentage (%) of trap sides with TuYV-carrying aphids explained 88% of variation (*p* < 0.001), % of trap sides with GPA 53% (*p* < 0.001) and aphid numbers per trap 51% (*p* < 0.001). Each multiple linear regression combination of these was insignificant, except the relationship between % of trap sides with TuYV-carrying aphids and the significant interaction between it and % of trap sides with GPA which explained 92% of variation (*p* < 0.001). When incorporated individually into a linear regression with final crop TuYV incidence as the dependent variable, % of trap sides with TuYV-carrying aphids explained 75% of variation (*p* < 0.001, Figure 3), % of trap sides with GPA 47% (*p* < 0.001), and aphid numbers per trap side 20% (*p* = 0.02). Each multiple linear regression combination of these was insignificant, except the relationship between % of trap sides with TuYV-carrying aphids and the significant interaction between it and % of trap sides with GPA which explained 76% of variation (*p* < 0.001).

## 3. Discussion

By collecting aphid trap and crop TuYV incidence data at 30 sites sown with canola over two years in the south-west Australian grainbelt, this study validated the in-field capability of a RT-LAMP assay protocol designed to detect TuYV in aphids. Furthermore, it demonstrated its application to virus disease management and epidemiological research. Using RT-PCR detection in total RNA extractions as the standard, the RT-LAMP assay accurately detected TuYV in pooled samples of winged aphids caught on double-sided yellow sticky traps. TuYV-carrying aphid detection was a strong predictor for subsequent virus spread in the crop. In all scenarios in which TuYV-carrying aphids were detected on >30% of trap sides over a six-week period from pre-emergence until GS15, TuYV reached >60% crop incidence by GS30. Conversely, TuYV detection on ≤15% trap sides during this period was associated with ≤6% TuYV incidence. Although the presence of aphids during this period was a prerequisite for spread to occur, there were multiple scenarios in which aphids were caught regularly, including GPA, but no TuYV detected in them, and minimal subsequent crop TuYV incidence at GS30. Furthermore, detection of GPA provided only minor, albeit inconsequential, benefit to epidemic prediction. Therefore, the protocol can provide early warning for TuYV epidemics and enable proactive disease management, predominantly non-prophylactic, more precisely timed and effective systemic insecticide applications. This early warning system developmental approach could be utilized for management of any externally sourced pathogen.

As demonstrated with other externally sourced viruses [29,31], this study demonstrated that the abundance of migrating viruliferous aphids in the environment is the most important direct epidemiological driver for TuYV spread in canola crops. Trapping and testing aphids for TuYV provided a strong and relevant estimation of background virus reservoir. In contrast, widespread sampling of broad-leaf weeds was resource intensive and rarely gave an indication of epidemic risk in the subsequent canola crop, likely because TuYV was below detection levels or reservoirs were further away. However, eliminating broad-leaf weed hosts at least two weeks prior to sowing (so aphids cannot migrate directly to the germinating crop) is still recommended to reduce TuYV inoculum as part of integrated disease management [32]. Aphid abundance *per se* was not as important, likely due to the presence of non-vector species and non-viruliferous GPA, as it is for internally sourced non-persistently transmitted viruses with a wide range of important vector species [33,34]. 

As observed when comparing South Stirlings and Coomalbidgup in 2018 (see Figure 1B,C), timing of viruliferous aphid flights was another critical epidemic driver. At South Stirlings, the crop was sown in mid-April in dry conditions with minimal broad-leaf weeds in the surrounding area. Despite this, TuYV-carrying aphids were caught regularly over a six-week period prior to germination, which was delayed due to lack of soil moisture. As there was no canola crop available to aphids for colonization, these flights ceased and TuYV spread in the young crop was avoided. In contrast, at Coomalbidgup, viruliferous aphid flights began prior to crop emergence but continued throughout the early growth stages. As a result, plants were colonized by GPA, primary TuYV infection foci formed and a pre-flowering epidemic eventuated. Indirectly, this comparison demonstrates the utility of delaying sowing. However, mid-autumn sowing is a well-subscribed practice in the region and delaying until late-autumn or early-winter can result in significant agronomic yield penalties [35]. If traps are deployed before sowing, the early warning system could be used to justify delaying sowing until autumn flights of migrant virus-carrying aphid flights have ceased, and recommend other control strategies such as application of a neonicotinoid seed treatment, stubble retention, high plant density delaying sowing [32]. 

The canola crops which experienced TuYV epidemics by GS30, likely incurred significant seed yield and quality losses [7,8]. Using the early warning system developed in this study, growers in these situations would be alerted to apply systemic insecticide (e.g., sulfoxaflor) to eliminate any initial GPA crop colonization, protect vulnerable plants from future infestations, and likely prevent epidemic level TuYV spread in pre-flowering canola and minimize subsequent seed yield and quality losses (illustrated in Figure 2). This insecticide application should be done (i) from GS15 if a well-applied neonicotinoid seed treatment has been used or (ii) immediately with untreated seed if TuYV has been detected in aphids and the crop has germinated [8,18]. However, research is required to understand, and thus predict, how environmental factors and metabolic resistance impact the efficacy of neonicotinoid seed treatments in the grainbelt so that the early warning system can be adapted and an informed intervention made with a foliar insecticide. Continued on-farm validation of the early warning system, involving testing crops that are sprayed with insecticide based on its recommendations, will ultimately determine its efficacy and further improve its application. 

The consistency of results within distinct grainbelt zones in this study suggests that testing of automated smart traps in a trapping network (currently being established in the region [36]) may provide enough data to get a reliable indication of area-wide virus risk. Additionally, this trapping program can be utilized to conduct surveillance for the R81T mutation. However, supplying yellow sticky traps and providing testing to grower advisors will give growers precise indications of TuYV risk in specific canola crops. Given the appropriate training and access to a portable LAMP machine, this protocol could be utilized by industry professionals, allowing them to test samples on site in a single consultation with the grower. Furthermore, this protocol is a faster and cheaper alternative to RT-PCR in a diagnostic laboratory. Once validated, similar early warning systems could enable surveillance of a wide range of disease-causing pathogens, e.g., other externally sourced viruses, and improve management of them.

## 4. Materials and Methods

### 4.1. Crude, Total RNA and Total DNA Extraction

For crude extraction of aphids for RT-LAMP, a polypropylene pellet pestle driven by a pellet pestle motor (Sigma-Aldrich, St. Louis, MO, USA) was used to grind aphids in a 1.5 mL tube containing 50 µL PBST buffer as described by [27]. Total RNA and DNA extraction was conducted from remaining crude extract (20 µL for each) using a QIAGEN RNeasy Plant Mini Kit and QIAamp 96 DNA QIAcube HT Kit, respectively, according to manufacturer instructions (QIAGEN, Chadstone, Victoria, Australia).

### 4.2. LAMP

All RT-LAMP or LAMP reactions were done using a dual-block (eight reaction wells per block) Genie^®^ II instrument (Optigene, Horsham, UK). In a total volume of 25 µL, the reaction mixture contained 3 µL (1:100 diluted crude aphid extraction) or 1 µL (total RNA/DNA extraction) template, 15 µL ISO-004 master mix (Optigene, Horsham, UK), 0.5 pmol each of F3 and B3, 2 pmol FIP and BIP and 1 pmol LF2 and LB2, 0.25 U of *Avian myeloblastosis virus* (AMV) reverse transcriptase (RNA only), and 2 µL RNase free water (total RNA/DNA extraction template only). The TuYV primer set TuYV4-ORF1 described by [27] was used (Table 3). For testing of crude and total RNA extractions, each set of eight reactions always included a negative and positive crude or total RNA/DNA extraction control in wells seven and eight, respectively. The reaction mixture was incubated at 65 °C for 40 min followed by an annealing step for 10 min. Results were analyzed in real-time via amplification and annealing graphs. A sample was considered positive if fluorescence exceeded 10,000 within the incubation time and annealing temperatures within 1 °C of those of positive controls. 

### 4.3. GPA-Specific LAMP Protocol Development and Validation

#### 4.3.1. Primer Design

A LAMP specific primer set (F3, B3, LF2, LB2, FIP and BIP) was derived from the GPA farnesyl diphosphate synthase 1 gene (accession no. EU429296) nucleotide sequence using PrimerExplorer V5 software (available at http://primerexplorer.jp/lampv5e/index.html) with default settings (Table 4). This primer set, GPA-FDS1, yielded an accurate, rapid and sensitive response to GPA DNA extractions. These were previously confirmed positive by morphological identification and sequencing (samples sent to Australian Genome Research Facility for Sanger Sequencing) of the mitochondrial cytochrome c oxidase 1 (CO1) gene amplified by PCR using GoTaq^®^ G2 Green Master Mix (Promega, Sydney, New South Wales, Australia) and primer set LepF (5’-TTCAACCAATCATAAAGATATTGG-3’) and LepR (5’-TAAACTTCTGGATGTCCAAAAAATCA-3’) [37]. 

#### 4.3.2. Specificity

Primer specificity was determined by testing the primer set against DNA extractions of other common grainbelt aphid species likely to be found on the yellow sticky traps: cabbage aphid, turnip aphid (*Lipaphis erysimi*), oat aphid (*Rhopalosiphum padi*), corn aphid (*Rhopalosiphum maidis*), blue-green aphid (*Acyrthosiphon kondoi*), melon aphid (*Aphis gossypii*) and cowpea aphid (*A. craccivora*). These were previously confirmed positive by morphological identification, and amplification and sequencing of the CO1 gene using primer set LepF and LepR. 

#### 4.3.3. Sensitivity

To test the sensitivity of the assay, a single GPA apterae was ground up individually or in groups of 9, 19, 49 and 99 cowpea aphids in 30 µL PBST buffer using a polypropylene pestle driven by a pellet pestle motor. The homogenate then underwent total DNA extraction. Each extraction was tested twice by LAMP and the experiment was repeated four times. 

### 4.4. RT-PCR

Two-step RT-PCR was performed to amplify the open reading frame (ORF) 3 RdRp gene nucleotide (nt) sequence of TuYV using primers TuYV1_3299F (5’-CGTAAGTTGCAAGTAAGGGAAAC-3’) and AS5 (5’-CCGGTTCYBCGTCTACCTATTTDG-3’) [27]. To obtain cDNA, reverse transcription was performed using an ImProm-II™ Reverse Transcription System with random primers (Promega, Australia). The cDNA was used to perform PCR amplification using GoTaq^®^ G2 Green Master Mix (Promega, Sydney, New South Wales, Australia) as done by [27].

### 4.5. ELISA

To test for TuYV infection in plant material, ELISA [38] was performed on leaf samples using *Beet western yellows virus* (BWYV) polyclonal antiserum (Sediag, Bretenière, France, cat. no. BWY-SRA 5000) as done by [27]. 

### 4.6. LAMP Protocol Field Validation

#### 4.6.1. Site Location

**Figure 4:**
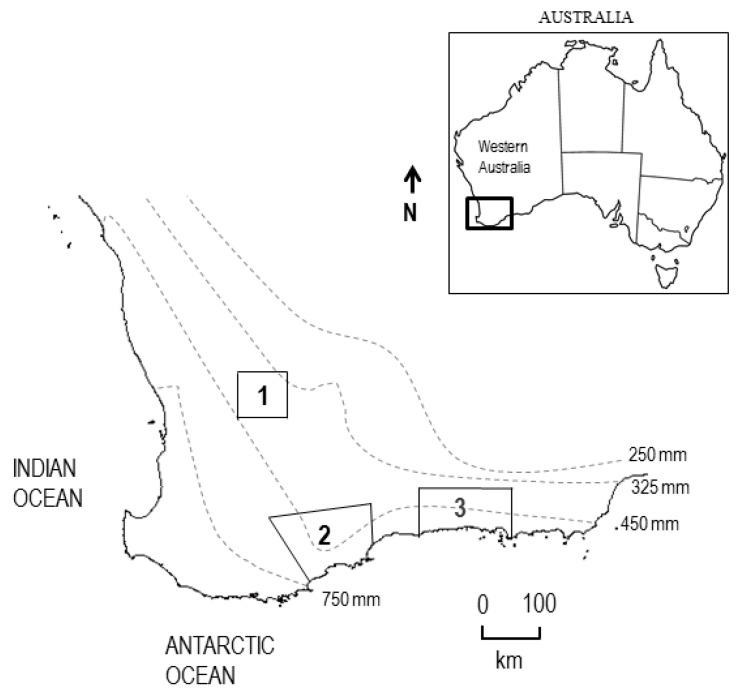
Map of Australia showing where the south-west Australian grain-growing region (grainbelt) is located. Insert shows the three zones in which loop-mediated isothermal amplification (LAMP) protocol field validation sites were located with 250 to 750 mm rainfall isohyets that makes the grainbelt boundaries.

In 2017 and 2018, field validation of the TuYV RT-LAMP protocol was undertaken at 30 farm sites (14 in 2017 and 16 in 2018) sown with canola in three distinct geographical zones in the south-west Australian grainbelt (Figure 4, Table 5). In both years, sites were located on farms in zone 1 near Nunile (31°50′ S, 116°54′ E) and Wongamine (31°46′ S, 116°49′ E); zone 2 near Gairdner (34°19′ S, 118°89′ E), Jerramungup (33°95′ S, 118°97′ E), Kendenup (34°53′ S, 117°60′ E), Mount Barker (34°61′ S, 117°71′ E) and South Stirlings (34°99′ S, 117°86′ E); and zone 3 near Coomalbidgup (33°44′ S, 121°19′ E), Esperance Downs (33°36′ S, 121°47′ E), Gibson (33°38′ S, 121°41′ E) and Munglinup (33°41′ S, 120°49′ E). In 2017, sites were also located on farms in zone 1 near Irish Town (31°57′ S, 116°62′ E); and zone 2 near Kojaneerup (34°56′ S, 118°29′ E) and Wellstead (34°47′ S, 118°66′ E). In 2018, sites were also located on farms in zone 1 near Bejoording (31°38′ S, 116°59′ E) and Coondle (31°48′ S, 116°41′ E); in zone 2 near Tenterdon (34°40′ S, 117°51′ E); and zone 3 near Dalyup (33°70′ S, 121°56′ E) and Grass Patch (33°23′ S, 121°54′ E).

#### 4.6.2. Aphid Trapping and Testing

At each site, three double-sided yellow sticky traps were tied to the top of the fence placed approximately 50 m apart along the fence line. Both sides of each trap were labelled to denote the side facing the canola crop and the side facing away. From approximately 3 to 12 weeks prior to sowing, traps were deployed and collected every two weeks. For 2017 traps, the aphids caught on each trap side were counted, extracted from the trap, placed in orange oil (De-Solv It, Vardon Industries, Australia) for 24 h to remove sticky glue, and then stored in 70% ethanol at 4 °C for up to seven months. Aphids then underwent crude extraction and were tested for TuYV by RT-LAMP, and then total RNA and DNA extraction were tested for TuYV and GPA by RT-PCR and RT-LAMP, respectively. The same method was used for 2018 traps, except that they were tested immediately. When testing for TuYV, ambiguous samples (i.e., confirmed positive by one method and not by the other) were tested again using total RNA extractions by RT-LAMP.

#### 4.6.3. Green-Bridge Host and Canola Crop Testing

To establish TuYV incidence in possible broad-leaf weed hosts prior to sowing, tip leaf samples (of up to 100 plants per species) were taken of afghan melon (*Citrullus lanatus*), blackberry nightshade (*Solanum nigrum*), clammy goosefoot (*Dysphania pumilio*), common sow thistle (*Sonchus oleraceus*), flaxleaf fleabane (*Conyza bonariensis*), marshmallow (*Malva palviflora*), serradella (*Ornithopus sativus*), soursob (*Oxalis pes-caprae*), subterranean clover, wild radish and volunteer canola and were tested in groups of 10 by ELISA. For canola crops, from approximately GS12 (two-leaf stage), crops were sampled every two to six weeks until GS75 (~50% podding) or when TuYV had reached 100% incidence in the crop. To do this, tip leaf samples of 100 to 200 plants were taken in a ‘W’ pattern from the fence line at the first trap to ~80 m diagonally into the crop, then diagonally back to the fence line at the second trap, and so on. All weed and crop samples were tested individually or in groups of two to 10 by ELISA. Virus incidence was estimated from grouped sample test results using the formula of Gibbs and Gower [39].

### 4.7. Statistical Analysis

For all statistical analysis, assumptions of normality and homogeneity of variance were checked using Shapiro test and through regression of residuals against fitted values, respectively, in all analyses. For GPA LAMP primer validation, differences in mean amplification time between each dilution were tested for significance using a one-way analysis of variance (ANOVA) and Tukey honest significant differences (HSD) test. 

To examine the relationships between data collected from sticky traps and subsequent crop TuYV incidence, linear regression and multiple linear regression were used. For the purposes of statistical analysis, all virus incidence data was angular transformed. For crop TuYV incidence, two data points were used at each site: (1) incidence at GS30 and (2) final incidence (incidence at end of exponential spread or final sampling). For (1), regression was performed with (i) mean aphid numbers per trap side, (ii) percent of trap sides with TuYV-carrying aphids and (iii) percent of trap sides with GPA, each assessed over a six-week period spanning pre-emergence until GS15 (five-leaf stage, ~six weeks prior to GS30). For (2), the same was done except each of (i), (ii) and (iii) was assessed in a four to twelve week period prior to final incidence. 

## Figures and Tables

**Table 1:** Detection of green peach aphids (GPA; *Myzus persicae*) individually or combined with groups of cowpea aphids (*Aphis craccivora*) by loop mediated isothermal amplification.

Dilution	Positives	Mean Time to Positive (min)
1/1 ^a^	4/4 ^b^	19.6 ± *0.9* ^c^
1/10	4/4	19.2 ± *0.7*
1/20	4/4	17.3 ± *0.6*
1/50	4/4	19.1 ± *0.5*
1/100	4/4	21.5 ± *0.7*
0/100	4/4	-
Negative	0/4	-
Positive	4/4	17.7 ± *0.5*

^a^ A single GPA apterae ground up individually or in groups of cowpea aphids in 30 uL PBST buffer with a polypropylene pestle driven by a pellet pestle motor, before undergoing total DNA extraction using a QIAamp 96 DNA QIAcube HT Kit according to manufacturer instructions. A 0/100 dilution is 100 cowpea aphids alone. ^b^ Number of repeat experiments TuYV-detected/total number of repeat experiments. ^c^ Time for fluorescence to exceed 10,000 within 40 min. Standard error in italics.

**Table 2:** *Turnip yellows virus* (TuYV), aphid number and green peach aphid (GPA; *Myzus persicae*) data collected at 30 sites sown to canola (*Brassica napus*) in the south-west Australian grainbelt in 2017 and 2018.

Location	Year	Zone ^a^	TuYV Detection in Broad-Leaf Weeds ^b^	Mean Aphids Per Trap Side Pre-Emergence to GS15 ^c^	Trap Sides with TuYV-Carrying Aphids Pre-Emergence to GS15 (%)	Trap Sides with GPA Pre-Emergence to GS15 (%)	Crop TuYV Incidence at GS30 (%) ^d^	Crop TuYV Incidence at GS75 (%)
Irish Town	2017	1	100% VC	5	32	32	60	100
Kojaneerup	2017	2	Nil	2	18	25	27	47
Coomalbidgup	2017	3	Nil	8	10	21	5	83
Jerramungup	2017	2	Nil	2	0	22	4	38
Kendenup	2017	2	Nil	2	0	9	4	27
Wongamine	2017	1	Nil	2	0	19	1	41
Munglinup	2017	3	Nil	4	0	25	1	27
South Stirlings	2017	2	<1% WR	2	4	0	1	25
Mount Barker	2017	2	Nil	5	0	29	1	23
Esperance Downs	2017	3	Nil	9	0	8	1	12
Nunile	2017	1	<1% WR	2	0	6	1	11
Gairdner	2017	2	Nil	3	0	0	0	75
Wellstead	2017	2	Nil	2	4	0	0	43
Gibson	2017	3	Nil	5	0	10	0	20
Esperance Downs	2018	3	Nil	7	32	39	88	100
Munglinup	2018	3	Nil	10	43	32	87	100
Gibson	2018	3	<1% SC	14	62	33	83	100
Dalyup	2018	3	-	11	58	33	83	100
Coomalbidgup	2018	3	Nil	11	67	14	79	100
Grass Patch	2018	3	Nil	6	54	29	62	100
Jerramungup	2018	2	Nil	1	15	15	6	63
Gairdner	2018	2	Nil	0	0	0	0	-
Nunile	2018	1	Nil	2	0	8	0	4
Wongamine	2018	1	Nil	1	0	0	0	4
South Stirlings	2018	2	Nil	1	0	0	0	3
Bejoording	2018	1	Nil	1	0	4	0	2
Mount Barker	2018	2	Nil	0	0	0	0	2
Coondle	2018	1	-	0	0	0	0	1
Tenterdon	2018	2	Nil	0	0	0	0	1
Kendenup	2018	2	Nil	0	0	0	0	0

^a^ See Figure 4. ^b^ If present before sowing, leaf samples taken from afghan melon (*Citrullus lanatus*), blackberry nightshade (*Solanum nigrum*), clammy goosefoot (*Dysphania pumilio*), common sow thistle (*Sonchus oleraceus*), flaxleaf fleabane (*Conyza bonariensis*), marshmallow (*Malva palviflora*), serradella (*Ornithopus sativus*), soursob (*Oxalis pes-caprae*), subterranean clover (SC; *Trifolium subterraneum*), wild radish (WR; *Raphanus raphanistrum*) and volunteer canola (VC) tested by enzyme-linked immunosorbent assay (ELISA).—denotes site not tested, nil denotes no virus detected. ^c^ Deployed three double-sided yellow sticky traps on top of fence line and collected every two weeks and total aphid numbers counted on each trap side. Aphids caught on each trap side were counted before being pooled, homogenized, and the crude extract tested for TuYV by reverse transcription loop-mediated isothermal amplification (LAMP). Half the remaining homogenate underwent total DNA extraction and then tested for GPA by LAMP. ^d^ Tip leaf samples of 200 plants taken from each canola crop and tested individually or in groups of 2 to 10 by ELISA,—denotes site not tested. Gibbs and Gower maximum likelihood estimator used to calculate percentage (%) incidence in grouped samples. Growth stages determined using the ‘BBCH’ decimal system: GS15—five-leaf stage, GS30—beginning of stem elongation, GS75—50% podding [4].

**Table 3:** Primer set TuYV4-ORF1 used for loop-mediated isothermal amplification of *Turnip yellows virus* (TuYV).

Primer	Type	Position on Genome ^a^	Length (nt)	Sequence 5′-3′
F3	Forward outer	897-914	18	TGATGTCACCCTCCTCCG
B3	Backward outer	1084-1102	19	AGTGTCCTCCTTCCGTGTG
FIP	Forward inner	970-991 and 926-945	42	TGCATTTTGCTAGGTTGGCAGCATTGGGAAGGACTGTTAGGC
BIP	Backward inner	1019-1040 and 1064-1083	42	ATGGCTGGGTTAGCGGTTATGCGCTCAGGACCATAACATCGG
LF2	Loop forward outer	946-964	19	TGACGTTGGCCGCTTTACA
LB2	Loop backward outer	1041-1062	22	CGAGATTGTAGGCTCAGAAGGT

**^a^** Genome position according to the reference nucleotide sequence of TuYV isolate WA-1 (ERS2791624) [27].

**Table 4:** Primer set GPA-FDS1 used for loop-mediated isothermal amplification of green-peach aphid (GPA; *Myzus persicae*) DNA.

Primer	Type	Position on Gene ^a^	Length (nt)	Sequence 5′-3′
F3	Forward outer	339-356	18	TACAGCCGTCAGCAAGGA
B3	Backward outer	534-553	20	CAGTCTGATCAGAAGGCGAG
FIP	Forward inner	406-426 and 362-281	41	TAAGTTACGGCCGGTGTCCGTCCAGGGATTTCATGGCAGTG
BIP	Backward inner	435-455 and 495-514	41	CGATGTTACCAAGTGGCCCGCGTACCAAAGCCAATCCTCGG
LF2	Loop forward outer	382-403	22	GATCCCTGACTACATCTGGGAA
LB2	Loop backward outer	456-479	24	AAAGCTGTTGCAATACAATGTGCC

^a^ Genome position according to the reference nucleotide sequence of GPA farnesyl diphosphate synthase 1 gene, accession no. EU429296.

**Table 5:** Canola (*Brassica napus*) growing site details for field validation of *Turnip yellows virus* (TuYV) reverse-transcription loop-mediated isothermal amplification protocol.

Year	Zone ^a^	Location	February to April Rainfall (mm)	Cultivar	Neonicotinoid Seed Treatment	Sowing Date	Date Aphid Trap First Deployed ^b^	Green-Bridge Species Tested (Number of Plants) ^c^
2017	1	Irish Town	123	ATR Bonito	No	1-May	8-Apr	VC (20)
2017	1	Nunile	123	InVigor T4510	Yes	16-May	8-Apr	AM (100), BN (100), CG (100), WR (100)
2017	1	Wongamine	123	Pioneer 43Y23	Yes	12-May	8-Apr	AM (100), CG (100), VC (100), WR (100)
2017	2	Gairdner	160	ATR Bonito	No	24-Apr	23-Mar	AM (100), BN (100), SC (100), Se (100), VC (100), WR (100)
2017	2	Jerramungup	131	ATR Bonito	Yes	20-Apr	23-Mar	BN (100), CG (100), SC (100), VC (100), WR (100)
2017	2	Kendenup	102	ATR Mako	Yes	8-May	23-Mar	BN (100), FF (100)
2017	2	Kojaneerup	137	ATR Mako	No	13-Jun	23-Mar	AM (100), BN (100), CG (100), SC (100), Se (100), VC (100), WR (100)
2017	2	Mount Barker	185	ATR Mako	Yes	1-May	23-Mar	Nil
2017	2	Wellstead	243	Thumper TT	Yes	20-Apr	23-Mar	BN (100), CG (100), Se (100), SC (100)
2017	2	South Stirlings	137	Nuseed GT-53	Yes	22-Apr	23-Mar	WR (100)
2017	3	Coomalbidgup	256	ATR Wahoo	Yes	25-Apr	30-Mar	BN (100), CG (100), VC (100), SC (100)
2017	3	Esperance Downs	221	ATR Mako	Yes	30-Apr	30-Mar	Se (100)
2017	3	Gibson	221	ATR Bonito	Yes	28-Apr	30-Mar	WR (100)
2017	3	Munglinup	256	Hyola 559TT	Yes	18-Apr	30-Mar	BN (100), WR (100)
2018	1	Bejoording	16	Nuseed GT-53	Yes	21-Apr	20-Mar	AM (3), WR (62)
2018	1	Coondle	16	ATR Bonito	Yes	14-May	10-Jul	-
2018	1	Nunile	16	Pioneer 44Y27	Yes	26-Apr	20-Mar	CST (10), So (100), WR (30)
2018	1	Wongamine	16	Pioneer 44Y27	Yes	25-Apr	20-Mar	So (50), WR (6)
2018	2	Gairdner	38	ATR Mako	No	10-Apr	27-Mar	Nil
2018	2	Jerramungup	42	ATR Bonito	No	10-Apr	27-Mar	AM (6), BN (2), CG (2), CST (6), FF (2), MM (15), SC (2), WR (8)
2018	2	Kendenup	39	InVigor T4510	Yes	23-May	27-Mar	AM (10), WR (15)
2018	2	Mount Barker	39	InVigor T4510	Yes	11-May	27-Mar	AM (10), SC (5), VC (20), WR (20)
2018	2	Tenterdon	36	InVigor T4510	Yes	3-May	27-Mar	Nil
2018	2	South Stirlings	44	Pioneer 45Y25	Yes	16-Apr	27-Mar	Nil
2018	3	Coomalbidgup	78	Hyola 404RR	Yes	5-May	27-Mar	AM (5), BN (3), CG (100), CST (16), WR (45)
2018	3	Dalyup	78	Pioneer 44Y27	Yes	15-May	19-Jun	-
2018	3	Esperance Downs	146	ATR Bonito	Yes	21-May	27-Mar	AM (4), SC (20), WR (7), VC (7)
2018	3	Gibson	146	ATR Bonito	Yes	28-Apr	27-Mar	MM (30), SC (65), WR (30),
2018	3	Grass Patch	186	ATR Bonito	No	7-Apr	27-Mar	MM (10), WR (18)
2018	3	Munglinup	78	Hyola 559TT	Yes	20-Apr	27-Mar	CST (10), VC (130), WR (95)

^a^ See Figure 4. ^b^ Three double-sided yellow sticky traps deployed on fence line and collected every two weeks. ^c^ If present, leaf samples taken from afghan melon (AM; *Citrullus lanatus*), blackberry nightshade (BN; *Solanum nigrum*), clammy goosefoot (CG; *Dysphania pumilio*), common sow thistle (CST; *Sonchus oleraceus*), flaxleaf fleabane (FF; *Conyza bonariensis*), marshmallow (MM; *Malva palviflora*), serradella (Se; *Ornithopus sativus*), soursob (So; *Oxalis pes-caprae*), subterranean clover (SC; *Trifolium subterraneum*), wild radish (WR; *Raphanus raphanistrum*) and volunteer canola (VC). Nil denotes no green-bridge material present,—denotes site not tested.

## References

[1] Australian Bureau of Statistics (2018). Value of Agricultural Commodities Produced.

[2] Seymour M., Kirkegaard J.A., Peoples M.B., White P.F., French R.J. (2012). Break-crop benefits to wheat in Western Australia—Insights from over three decades of research. Crop Pasture Sci..

[3] Coutts B.A., Jones R.A.C. (2000). Viruses infecting canola (*Brassica napus*) in south-west Australia: incidence, distribution, spread and infection reservoir in wild radish (*Raphanus raphinistrum*). Aust. J. Agric. Res..

[4] Lancashire P.D., Bleiholder H., Boom T.V.D., Langelüddeke P., Stauss R., Weber E., Witzenberger A. (1991). A uniform decimal code for growth stages of crops and weeds. Ann. Appl. Biol..

[5] Graichen K., Schliephake E. Infestation of winter oilseed rape by turnip yellows luteovirus and its effect on yield in Germany. Proceedings of the 10th International Rapeseed Congress—New Horizons for an Old Crop.

[6] Jay C.N., Rossall S., Smith H.G. (1999). Effects of beet western yellows virus on growth and yield of oilseed rape (*Brassica napus*). J. Agric. Sci..

[7] Jones R.A.C., Coutts B.A., Hawkes J.R. (2007). Yield-limiting potential of *Beet western yellows virus* in *Brassica napus*. Aust. J. Agric. Res..

[8] Congdon B.S., Matson P., Begum F., Dore A., Kehoe M.A., Coutts B.A. *Turnip yellows virus* epidemic in 2018—Time to get one step ahead of the green peach aphid. Proceedings of the 2019 GRDC Research Updates.

[9] Weber G. (1985). Genetic variability in host plant adaptation of the green peach aphid, Myzus persicae. Entomol. Exp. Appl..

[10] Mathers T.C., Chen Y., Kaithakottil G., Legeai F., Mugford S.T., Baa-Puyoulet P., Bretaudeau A., Clavijo B., Colella S., Collin O. (2017). Rapid transcriptional plasticity of duplicated gene clusters enables a clonally reproducing aphid to colonise diverse plant species. Genome Biol..

[11] Schliephake E., Graichen K., Rabenstein F. (2000). Investigations on the vector transmission of the *Beet mild yellowing virus* (BMYV) and the *Turnip yellows virus* (TuYV). J. Plant Dis. Protect..

[12] Anstead J.A., Williamson M.S., Denholm I. (2005). Evidence for multiple origins of identical insecticide resistance mutations in the aphid *Myzus persicae*. Insect Mol. Biol..

[13] Umina P.A., Edwards O., Carson P., Van Rooyen A., Anderson A. (2014). High levels of resistance to carbamate and pyrethroid chemicals widespread in Australian *Myzus persicae* (Hemiptera: Aphididae) populations. J. Econ. Entomol..

[14] Severtson D., Flower K., Nansen C. (2015). Nonrandom distribution of cabbage aphids (Hemiptera: Aphididae) in dryland canola (Brassicales: Brassicaceae). Environ. Entomol..

[15] Coutts B.A., Hawkes J.R., Jones R.A.C. (2006). Occurrence of *Beet western yellows virus* and its aphid vectors in over-summering broad-leafed weeds and volunteer crop plants in the grainbelt region of south-western Australia. Aust. J. Agric. Res..

[16] Latham L.J., Smith L.J., Jones R.A.C. (2003). Incidence of three viruses in vegetable brassica plantings and wild radish weeds in south-west Australia. Australas. Plant Pathol..

[17] Maling T., Diggle A.J., Thackray D.J., Siddique K.H.M., Jones R.A.C. (2010). An epidemiological model for externally acquired vector-borne viruses applied to *Beet western yellows virus* in *Brassica napus* crops in a Mediterranean-type environment. Crop Pasture Sci..

[18] Coutts B.A., Webster C.G., Jones R.A.C. (2010). Control of *Beet western yellows virus* in *Brassica napus* crops: Infection resistance in Australian genotypes and effectiveness of imidacloprid seed dressing. Crop Pasture Sci..

[19] Sekulic G., Rempel C.B. (2016). Evaluating the role of seed treatments in canola/oilseed Rape production: integrated pest management, pollinator health, and biodiversity. Plants.

[20] Stamm M.D., Heng-Moss T.M., Baxendale F.P., Siegfried B.D., Blankenship E.E., Nauen R. (2016). Uptake and translocation of imidacloprid, clothianidin and flupyradifurone in seed-treated soybeans. Pest Manag. Sci..

[21] De Little S.C., Edwards O., van Rooyen A.R., Weeks A., Umina P.A. (2017). Discovery of metabolic resistance to neonicotinoids in green peach aphids (*Myzus persicae*) in Australia. Pest Manag. Sci..

[22] Sparks T.C., Watson G.B., Loso M.R., Geng C., Babcock J.M., Thomas J.D. (2013). Sulfoxaflor and the sulfoximine insecticides: Chemistry, mode of action and basis for efficacy on resistant insects. Pestic. Biochem. Phys..

[23] Annetts R., Elias N., Corr I. Transform™ Insecticide (sulfoxaflor) for control of aphids in canola. Proceedings of the 16th Agronomy Conference 2012.

[24] Bass C., Puinean A.M., Andrews M., Cutler P., Daniels M., Elias J., Paul V.L., Crossthwaite A.J., Denholm I., Field L.M. (2011). Mutation of a nicotinic acetylcholine receptor β subunit is associated with resistance to neonicotinoid insecticides in the aphid *Myzus persicae*. BMC Neurosci..

[25] Bass C., Denholm I., Williamson M.S., Nauen R. (2015). The global status of insect resistance to neonicotinoid insecticides. Pestic. Biochem. Phys..

[26] Bass C., Puinean A.M., Zimmer C.T., Denholm I., Field L.M., Foster S.P., Gutbrod O., Nauen R., Slater R., Williamson M.S. (2014). The evolution of insecticide resistance in the peach potato aphid, *Myzus persicae*. Insect Biochem. Mol. Biol..

[27] Congdon B.S., Kehoe M.A., Filardo F.F., Coutts B.A. (2019). In-field capable loop-mediated isothermal amplification detection of *Turnip yellows virus* in plants and its principal aphid vector *Myzus persicae*. J. Virol. Methods.

[28] Fabre F., Kervarrec C., Mieuzet L., Riault G., Vialatte A., Jacquot E. (2003). Improvement of *Barley yellow dwarf virus*-PAV detection in single aphids using a fluorescent real time RT-PCR. J. Virol. Methods.

[29] Marroquín C., Olmos A., Teresa Gorris M.A., Bertolini E., Carmen Martínez M., Carbonell E.A., Hermoso de Mendoza A., Cambra M. (2004). Estimation of the number of aphids carrying *Citrus tristeza virus* that visit adult citrus trees. Virus Res..

[30] Cambra M., Bertolini E., Olmos A., Capote N., Cooper J.I., Kuehne T., Polischuk V. (2006). Molecular methods for detection and quantitation of virus in aphids. Virus Disease and Crop Biosecurity.

[31] Fabre F., Plantegenest M., Mieuzet L., Dedryver C.A., Leterrier J.-L., Jacquot E. (2005). Effects of climate and land use on the occurrence of viruliferous aphids and the epidemiology of barley yellow dwarf disease. Agric. Ecosyst. Environ..

[32] Jones R.A.C. (2006). Control of plant virus diseases. Adv. Virus Res..

[33] Maling T., Diggle A.J., Thackray D.J., Siddique K.H.M., Jones R.A.C. (2008). An epidemiological model for externally sourced vector-borne viruses applied to *Bean yellow mosaic virus* in lupin crops in a Mediterranean-type environment. Phytopathology.

[34] Thackray D.J., Diggle A.J., Berlandier F.A., Jones R.A.C. (2004). Forecasting aphid outbreaks and epidemics of *Cucumber mosaic virus* in lupin crops in a Mediterranean-type environment. Virus Res..

[35] Farré I., Robertson M.J., Walton G.H., Asseng S. (2002). Simulating phenology and yield response of canola to sowing date in Western Australia using the APSIM model. Aust. J. Agric. Res..

[36] Severtson D., Congdon B.S., Valentine C. Apps, traps and LAMP’s: ‘Smart’ improvements to pest and disease management. Proceedings of the 2018 Grains Research Updates.

[37] Coeur d’acier A., Cruaud A., Artige E., Genson G., Clamens A.-L., Pierre E., Hudaverdian S., Simon J.-C., Jousselin E., Rasplus J.-Y. (2014). DNA barcoding and the associated PhylAphidB@se website for the identification of european aphids (Insecta: Hemiptera: Aphididae). PLoS ONE.

[38] Clark M.F., Adams A.N. (1977). Characteristics of the microplate method of enzyme-linked immunosorbent assay for the detection of plant viruses. J. Gen. Virol..

[39] Gibbs A.J., Gower J.C. (1960). The use of a multiple-transfer method in plant virus transmission studies—Some statistical points arising in the analysis of results. Ann. Appl. Biol..

